# Prospective analysis of adiposity metrics for complicated acute colonic diverticulitis: Should WSES Classification and visceral adiposity be integrated for predictive analyses?

**DOI:** 10.1007/s00068-025-02884-w

**Published:** 2025-05-19

**Authors:** Damla Beyazadam, Tunc Eren, Nuray Colapkulu Akgul, Sukriye Julide Sagiroglu, Nesrin Gunduz, Ozgur Ekinci, Orhan Alimoglu

**Affiliations:** 1 Department of General Surgery, University of Health Sciences, Haydarpasa Numune Training and Research Hospital, Istanbul, Türkiye; 2 Department of General Surgery, Istanbul Medeniyet University, Goztepe Prof. Dr. Suleyman Yalcin City Hospital, Istanbul, Türkiye; 3 Department of General Surgery, Kocaeli Gebze Fatih State Hospital, Kocaeli, Türkiye; 4 Department of General Surgery, NMC Royal Hospital, Sharjah, United Arab Emirates; 5 Department of Radiology, Istanbul Medeniyet University, Goztepe Prof. Dr. Suleyman Yalcin City Hospital, Istanbul, Türkiye

**Keywords:** Complicated diverticulitis, Obesity, Visceral adiposity, Body mass index, WSES

## Abstract

**Background:**

Colonic diverticulitis is correlated with age, body mass index (BMI), and increased visceral or subcutaneous fat. Obesity contributes to diverticulosis, diverticulitis and its complications onset, as visceral fat activates inflammatory pathways that exacerbate these conditions. The aim of this study was to evaluate the association of visceral adiposity and BMI on complications of acute colonic diverticulitis.

**Method:**

A prospective cohort study was conducted on patients with acute diverticulitis admitted to the general surgery ward of a university hospital in Istanbul from September 2017 to February 2022. A total of 208 patients were classified into uncomplicated and complicated diverticulitis per the World Society of Emergency Surgery guidelines. After documenting demographic, clinical, and laboratory data, along with BMI and computed tomography measurements of visceral and subcutaneous fat areas (VFA, SFA), we conducted a statistical analysis to assess the relationships between VFA, SFA, and BMI in both groups.

**Results:**

Complicated diverticulitis group was found to have significantly lower values of BMI and VFA (*p* = 0.037 and *p* = 0.046 respectively). Similarly, BMI and VFA values of patients who underwent all interventional procedures including percutaneous drainage and surgery were found to be significantly lower than the conservative treatment group (*p* = 0.007, *p* = 0.025 respectively). V/S < 0.68 is an independent predictor in the complicated group.

**Conclusion:**

Our study shows that complicated diverticulitis cases have lower BMI and visceral fat area. We suggest that increased visceral adipose tissue may serve a protective role by preventing these diverticula from developing into more complicated forms. Therefore, visceral adiposity metrics can be integrated in the predictive analyses.

## Introduction

Diverticular disease of the colon is a common condition and the prevalence of diverticulosis ranges from 5 to 45% and increases with age [1–3]. Individuals with diverticulosis have 4% to 25% risk of experiencing diverticulitis at least once in their lifetime and an increase in the overall incidence of diverticulitis has been observed in the last two decades [4–7]. A study conducted in the United States revealed that hospitalization rates for diverticulitis admissions rose by 26% over the span of eight years. Notably, this increase was more pronounced among patients aged 18 to 44 years, with an increase of 82%, and among those aged 45 to 74 years, where the rate rose by 36% [8].

In recent years, management of diverticular disease and its complications have changed significantly, and conservative methods gained importance with new guidelines [9]. In 2015, the World Society of Emergency Surgery (WSES) presented a more comprehensive alternative to the widely used Hinchey classification, carefully encompassing both complicated and uncomplicated diverticulitis. Additionally, in 2016, they introduced a detailed diagnostic and therapeutic algorithm of acute diverticulitis. According to this classification based on computed tomography (CT) findings, diverticulitis was divided into two groups: complicated and uncomplicated diverticulitis. Uncomplicated diverticulitis was defined as the sole involvement of the colon and infection does not extend to the peritoneum. In complicated diverticulitis infectious process extends beyond the colon to distant localizations [10, 11].

Colon diverticular disease is increasingly common, yet the factors contributing to its pathogenesis and complications remain unclear [8, 12, 13]. Obesity is identified as a risk factor for diverticulitis, with literature indicating a link between obesity, colonic diverticulosis, and its complications [13–19]. Higher body mass index (BMI) values in recurrent and complicated diverticulitis cases, including perforation or abscess, compared to diverticulosis were shown [14]. Incidence of diverticulitis and related complications are therefore associated with increased visceral or subcutaneous fat [16, 20–23]. Visceral and subcutaneous adipose tissues exhibit different properties regarding bioactive molecule production, with visceral adipose tissue generating more inflammatory cytokines such as IL-6 and TNF-alpha. Obese individuals tend to have lower levels of adiponectin, an anti-inflammatory molecule, which is more abundant in visceral fat than in total fat. This has prompted investigations into the link between visceral fat and complicated diverticulitis [24, 25].

CT in determining visceral adiposity is significant. It has been shown that visceral fat measurement is more effective than traditional obesity markers like BMI and waist circumference measurement [19, 22, 26, 27]. Although certain studies in the literature suggest that values of visceral fat area (VFA) and BMI are lower in cases of complicated diverticulitis in comparison to uncomplicated cases, definitive conclusions cannot yet be established, as other studies present contradictory results or indicate no significant differences [19, 22, 26–28].

The primary endpoint of this study is to assess the prognostic significance of visceral adiposity, specifically VFA, the V/S ratio, and BMI, in relation to complicated diverticulitis as defined by the WSES CT-based staging system. The secondary endpoints are to identify inflammatory markers (such as C-Reactive Protein (CRP), procalcitonin, White Blood Cell (WBC) count, and neutrophil count), disease localization, and demographic variables that may predict disease severity. Additionally, our study aims to detail the clinical progression, imaging-based interventions including drainage and surgical procedures, and short-term outcomes such as duration of hospital stay, the necessity for surgical intervention and recurrence in patients diagnosed with complicated diverticulitis.

## Method

**Fig. 1:**
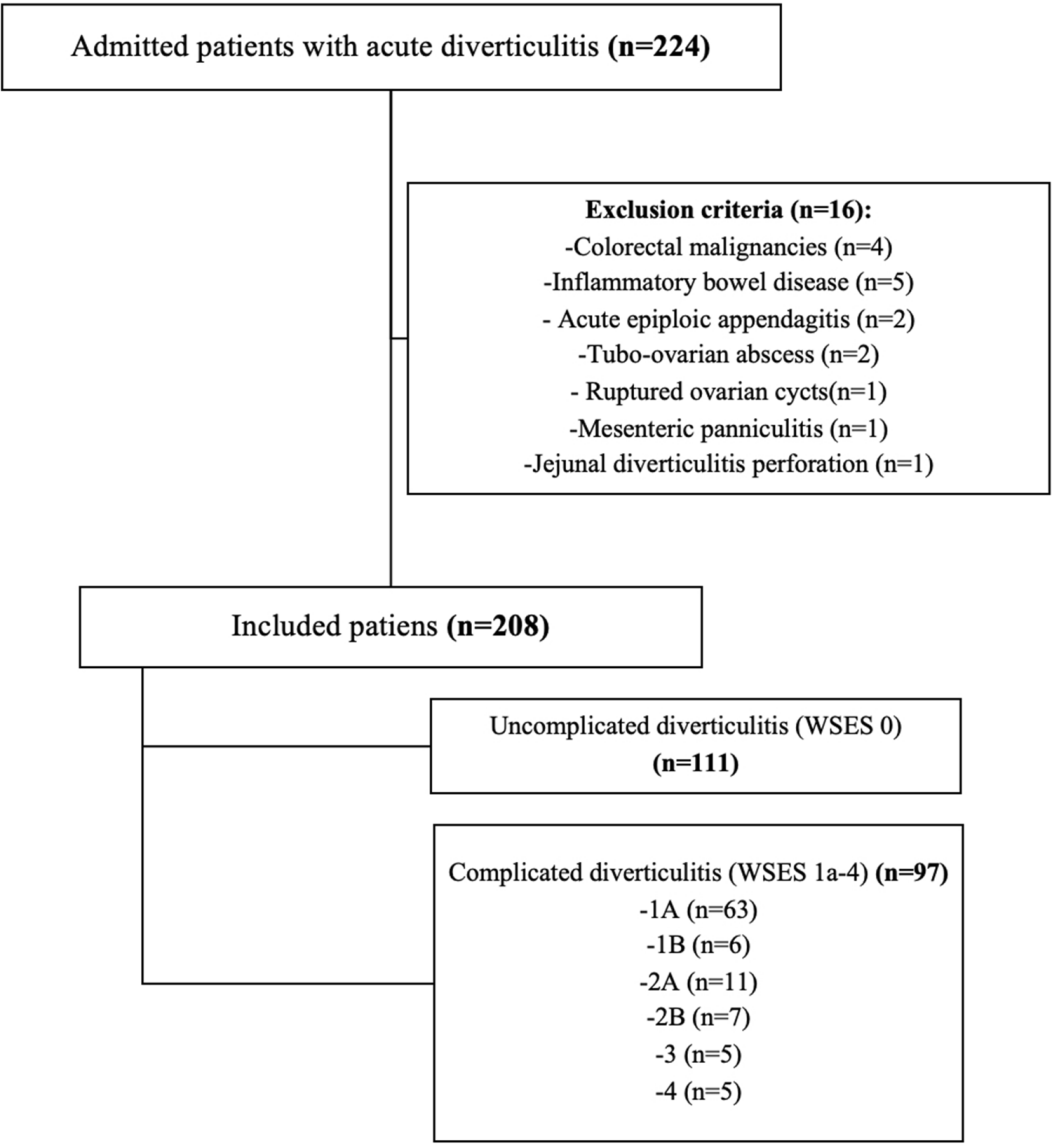
Patient Population

The study was designed as a prospective observational cohort and conducted between September 2017 and February 2022 at a university hospital in Istanbul. The study was approved by the hospital’s Ethics Committee with the approval number 2021/0223. Written informed consent was obtained from participants prior to the study. Patients presenting to the emergency department with abdominal pain and subsequently diagnosed with acute colonic diverticulitis via intravenous contrasted CT were admitted to the general surgery ward. These individuals underwent a thorough evaluation for inclusion in the study. Exclusion criteria were: 1) under the age of 18, 2) pregnancy, 3) patients with diagnosis of any inflammatory bowel disease, 4) CT scan with inflammatory bowel disease findings, 5) concomitant colon cancer (Fig. 1).

After being admitted in surgical ward, patients’ demographic features, comorbidities, weight, height, nutritional habits and bowel habits were questioned and recorded. Previous diverticulitis attacks were questioned if present. Patients’ physical examination findings and vital signs were recorded. Complete blood count, biochemical parameters, CRP and procalcitonin levels upon admission were also obtained.

Patients were divided into two groups as Uncomplicated group and Complicated group based on CT findings regarding WSES stages: 1) Uncomplicated (Stage 0: Diverticula, thickening of the wall, increased density of the pericolic fat), 2) Complicated [Stage 1 A: Pericolic air bubbles or small amount of pericolic fluid without abscess (within 5 cm from inflamed bowel segment), Stage1B: Abscess ≤ 4 cm, Stage 2 A: Abscess > 4 cm, Stage 2B: Distant gas (> 5 cm from inflamed bowel segment), Stage 3: Diffuse fluid without distant free gas, Stage 4: Diffuse fluid with distant free gas]. The methodology employed at our clinic involved the hospitalization of all patients with diverticulitis, including those without complications, and the administration of antibiotic therapy from 2017 to 2022, during which the study was conducted. Consequently, the research protocol was developed in accordance with this approach.

All patients underwent bowel rest, received intravenous antibiotics (3. generation cephalosporine and metronidazole combination), intravenous fluids and analgesics. Intravenous antibiotics were continued throughout the hospitalization period. Patients showing clinical improvement based on follow-up examinations on the 2nd or 3rd day of hospitalization were started on oral intake of fluids and soft foods. Patients with deteriorated clinical and laboratory findings were reevaluated through imaging, and their treatments were modified according to complications. The guidelines advocate for antibiotic treatment alone for abscesses smaller than 4 cm; however, they stipulate that in instances of no regression or clinical deterioration after 4–6 days of follow-up, percutaneous drainage should be undertaken alongside radiological re-evaluation [11]. Consequently, drainage was performed for abscesses exceeding 4 cm, and antibiotic therapy was adjusted based on the culture results of the abscess material. Surgical interventions were conducted if no regression was observed in the control imaging performed on the 4 th day and if clinical evaluation indicated deterioration. Emergency surgery was performed for patients with diffuse peritonitis, open perforation, obstruction, fistula to adjacent structures, septic shock, those who did not respond to conservative treatment. The decision for elective surgery was made considering conditions such as recurrent diverticulitis attacks, persistent abdominal pain, and development of complications. Upon discharge, patients were prescribed oral antibiotics and scheduled for a follow-up appointment in the first week. Interval colonoscopy was performed 4–6 weeks after discharge for patients who had not undergone colonoscopy prior 6 months to admission to rule out malignancy.

**Fig. 2:**
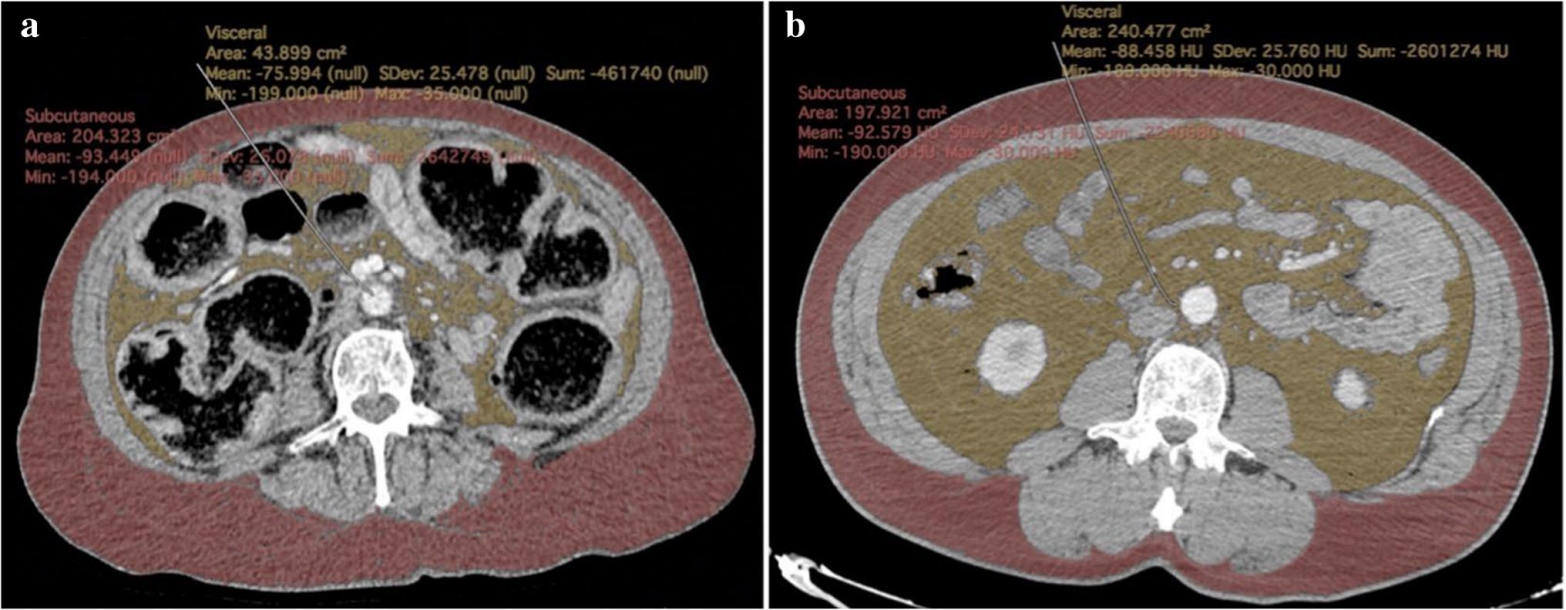
**a** Measurement of subcutaneous and visceral fat areas in axial computed tomography sections obtained at the L3-L4 vertebral level in a case of complicated diverticulitis presented with obstruction. **b** Measurement of subcutaneous and visceral fat area in axial computed tomography sections obtained from the L3-L4 vertebral level in a case of uncomplicated diverticulitis

The CT images of the patients were examined to measure visceral and subcutaneous fat areas. Horos DICOM software version 3.3.6 which is a open-source medical image viewer was used for radiological adiposity measurements. The CT attenuation range was determined as −190 to −30 Hounsfield units. VFA and Subcutaneous fat area (SFA) were measured by obtaining a single axial section at the level of the L3-L4 vertebrae, which were specified as reference levels in previous studies [13, 22]. The boundaries of VFA (cm^2^) and SFA (cm^2^) were manually defined and VFA/SFA (V/S) ratio was calculated (Fig. 2).

### Statistical analyses

**Fig. 3:**
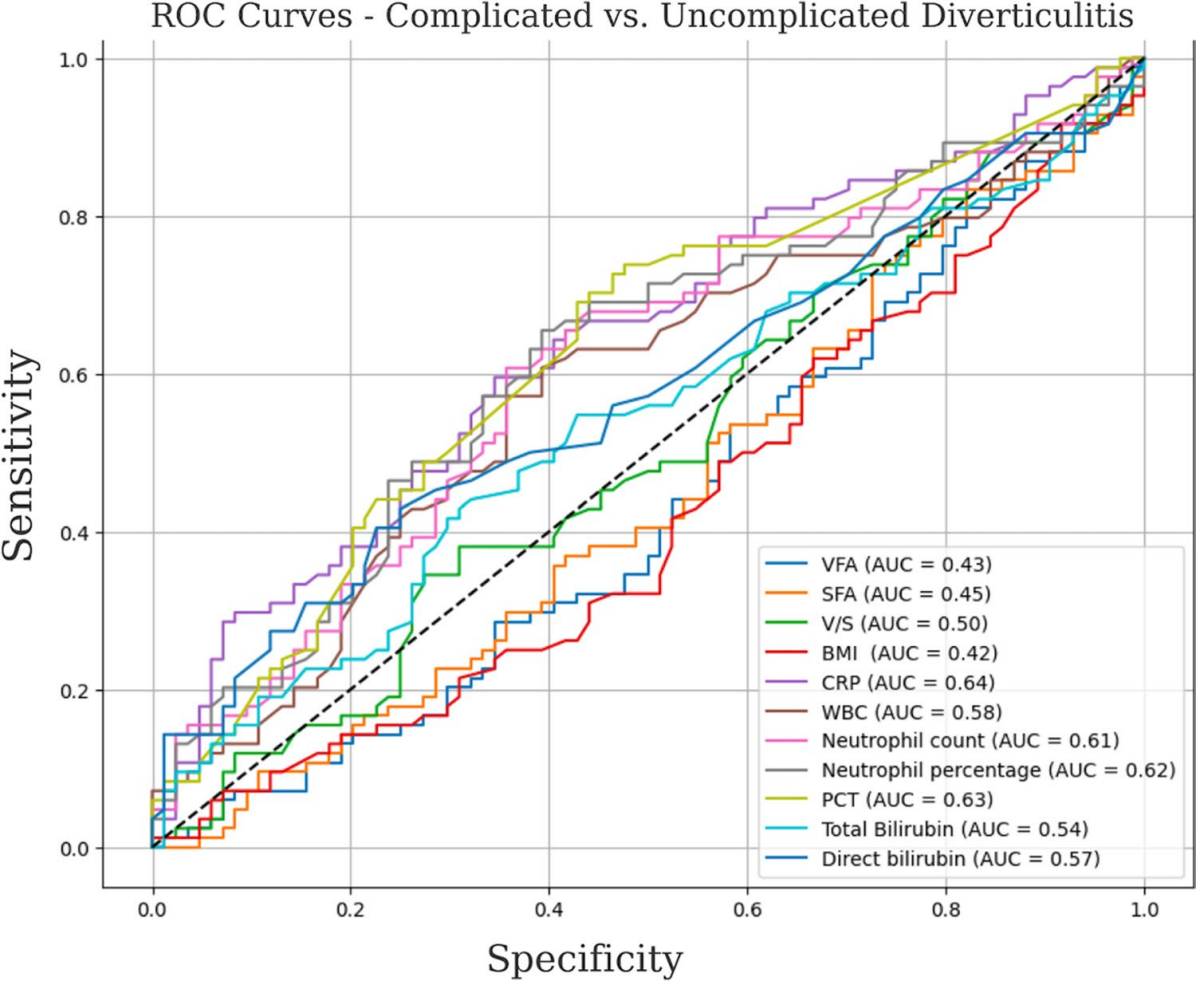
ROC Analysis of Biomarkers and Adiposity Metrics in Diverticulitis

Statistical analyses were conducted using SPSS (Statistical Package for the Social Sciences) version 20 (SPSS Inc.; Chicago, IL, USA). Descriptive statistics were presented as number and percentage for categorical variables, and as mean ± standard deviation or median (minimum–maximum value) for continuous variables. The presence of normal distribution was determined using the Shapiro–Wilk test. For categorical variables, comparisons were made using Pearson's chi-square test or Fisher's exact test when assumptions were not met. In tables larger than 2 × 2 where the number of expected values was < 5, the Fisher-Freeman-Halton test was used. When comparing continuous variables between two groups, independent samples t-test or Mann–Whitney U test was used depending on whether normal distribution was present. For comparison of independent three groups, one-way analysis of variance (ANOVA) was used if normal distribution was present, otherwise the Kruskal- Wallis test was used. Cases where the two-tailed p-value was < 0.05 were considered statistically significant. Receiver Operating Characteristic (ROC) curve analysis was performed to evaluate the diagnostic performance of inflammatory markers (CRP, Procalcitonin, WBC count, neutrophil percentage) and adiposity measures VFA, SFA, V/S, and BMI in patients with diverticulitis. The area under the curve (AUC) and 95% confidence intervals (CIs) were calculated, with optimal cut-off values identified via the Youden Index. An AUC > 0.7 was considered acceptable. CRP showed the highest diagnostic accuracy (AUC = 0.812), followed by Procalcitonin (AUC = 0.789). Adiposity metrics showed moderate predictive value. A p-value < 0.05 was considered statistically significant (Fig. 3).

Univariable Cox proportional hazards regression analyses identified the relationship between clinical, laboratory, and radiologic variables and complicated diverticulitis occurrence. The “duration” variable was standardized across patients to estimate hazard ratios within a cross-sectional framework. Variables with p-values less than 0.1 in univariable analysis or deemed clinically significant were included in a multivariable Cox regression model using forward stepwise selection. Hazard ratios, 95% confidence intervals (CI), and two-sided p-values were calculated, with statistical significance defined as *p* < 0.05. Multicollinearity was evaluated with variance inflation factors, and model assumptions were assessed with Schoenfeld residuals.

## Results

The study included 208 patients, of which 109 were female patients (52.4%) and 99 were male (47.6%), with a mean age of 57.2 (± 15.2) years. Uncomplicated group comprised 53.4% (*n* = 111) while complicated group comprised 46.6% (*n* = 97) (Table 1).

**Table 1:** Comparison of Demographics, Laboratory Findings, Clinical Course, Treatment, and Follow-up Parameters of Complicated and Uncomplicated Diverticulitis

			All patients	Uncomplicated	Complicated	*p*
				(WSES 0)	(WSES 1a-4)	
			*n* = 208	*n* = 111 (53.49%)	*n* = 97 (46.6%)	
Age (years)						0.327a
		*Mean* ± *SD*	57.2 ± 15.2	56.3 ± 15.3	58.3 ± 15	
		*Median (min.–max.)*	57 (22–87)	56 (22–86)	58 (24–87)	
Gender						> 0.999b
	Male	*n (%)*	99 (47.6%)	53 (47.7%)	46 (47.4%)	
	Female	*n (%)*	109 (52.4%)	58 (52.3%)	51 (52.6%)	
CRP* (mg/dL)						< 0.001b**
		*Mean* ± *SD*	8.28 ± 8.42	6.27 ± 7.02	10.5 ± 9.29	
		*Median (min.–max.)*	6.1 (0.1–45.6)	4.62 (0.1–45.6)	7.9 (0.1–42.3)	
WBC** (× 10^3^/μL)						0.028b*
		*Mean* ± *SD*	12.72 ± 4.04	12.13 ± 3.38	13.39 ± 4.61	
		*Median (min.–max.)*	12.1 (5.1–41)	11.6 (5.1–25.6)	12.7 (5.6–41)	
Neutrophil count (× 10^3^/μL)						0.003b**
		*Mean* ± *SD*	9.36 ± 3.4	8.69 ± 3.14	10.12 ± 3.54	
		*Median (min.–max.)*	8.98 (2.5–22)	8.06 (2.5–22)	9.73 (3.6–21.35)	
Neutrophil percantage (%)						0.001b**
		*Mean* ± *SD*	72.2 ± 12.1	70.5 ± 10.1	74.3 ± 13.8	
		*Median (min.–max.)*	73.8 (8.3–93.6)	70.3 (42.8–90.8)	76.2 (8.3–93.6)	
Procalcitonin (ng/mL)						0.005b**
		*Mean* ± *SD*	1.67 ± 10.5	0.29 ± 1.17	3.12 ± 14.9	
		*Median (min.–max.)*	0.1 (0.02–117)	0.07 (0.02–11)	0.1 (0.03–117)	
Direct Bilirubin (mg/dL)						0.020b*
		*Mean* ± *SD*	0.29 ± 0.19	0.26 ± 0.15	0.33 ± 0.23	
		*Median (min.–max.)*	0.24 (0.09–1.21)	0.22 (0.09–0.88)	0.27 (0.09–1.21)	
Number of Episodes						
	Single episode	*n (%)*	140 (67.3%)	81 (73.0%)	59 (60.8%)	0.086c
	Multiple episodes	*n (%)*	68 (32.7%)	30 (27.0%)	38 (39.2%)	
Family history						
	No	*n (%)*	191 (91.8%)	102 (91.9%)	89 (91.8%)	> 0.999c
	Yes	*n (%)*	17 (8.17%)	9 (8.11%)	8 (8.25%)	
Localization						
	Sigmoid Colon	*n (%)*	154 (74.0%)	74 (66.7%)	80 (82.5%)	0.005 d**
	Descending Colon	*n (%)*	19 (9.13%)	14 (12.6%)	5 (5.15%)	
	Transverse Colon	*n (%)*	3 (1.44%)	0 (0.00%)	3 (3.09%)	
	Ascending Colon	*n (%)*	9 (4.33%)	8 (7.21%)	1 (1.03%)	
	Cecum	*n (%)*	23 (11.1%)	15 (13.5%)	8 (8.25%)	
Obstruction						
	No	*n (%)*	203 (97.6%)	109 (98.2%)	94 (96.9%)	0.666e
	Yes	*n (%)*	5 (2.40%)	2 (1.80%)	3 (3.09%)	
Fistula						
	No	*n (%)*	205 (98.6%)	111 (100%)	94 (96.9%)	0.100e
	Yes	*n (%)*	3 (1.44%)	0 (0.00%)	3 (3.09%)	
Treatment						
	Conservative	*n (%)*	177 (85.1%)	108 (97.3%)	69 (71.1%)	< 0.001c**
	Invasive	*n (%)*	31 (14.9%)	3 (2.70%)	28 (28.9%)	
Invasive procedure						
	No	*n (%)*	177 (85.1%)	108 (97.3%)	69 (71.1%)	< 0.001 d**
	Percutaneous drainage	*n (%)*	7 (3.37%)	0 (0.00%)	7 (7.22%)	
	Emergency surgery	*n (%)*	13 (6.25%)	1 (0.90%)	12 (12.4%)	
	Elective surgery	*n (%)*	8 (3.85%)	2 (1.80%)	6 (6.19%)	
	Percutaneous drainage and emergency surgery	*n (%)*	3 (1.44%)	0 (0.00%)	3 (3.09%)	
Follow-up period (month)						
		*Mean* ± *SD*	30.7 ± 14.8	28.5 ± 13.6	33.2 ± 15.9	0.026b*
		*Median (min.–max.)*	30 (6–61)	30 (6–56)	32.5 (6–61)	

*Abbreviations: CRP *C-reactive protein, *WBC *White blood cell count

a: Independent Variable test, b: Mann Whitney -U test, c: Pearson chi-square test :, d: Fisher freeman Halton test, e:Fisher's exact test

*p<0.05, **p<0.01

When clinical and laboratory findings were compared, CRP, WBC count, neutrophil count, neutrophil percentage, procalcitonin, and direct bilirubin were found to be significantly higher in the complicated diverticulitis group compared to the uncomplicated group (*p* < 0.001, *p* = 0.028, *p* = 0.003, *p* = 0.001, *p* = 0.005, *p* = 0.02, respectively) (Table 1). There were no significant differences in vital signs between the groups.

The overall mean follow-up period was 30.7 ± 14.8 (6–61) months, with the complicated group at 33.2 ± 15.9 months, significantly longer than the uncomplicated group at 28.5 ± 13.6 months (*p* = 0.026). Additionally, 32.7% of patients had a previous history of multiple episodes, while 67.3% had a single episode. There were no statistically significant difference in number of episodes between uncomplicated and complicated groups. Among the cohort, 154 individuals (74%) exhibited involvement of the sigmoid colon, whereas 23 patients (11.1%) were affected in the cecum, 19 patients (9.13%) in the descending colon, 9 patients (4.33%) in the ascending colon, and 3 patients (1.44%) in the transverse colon (Table 1). Sigmoid colon involvement was significantly more frequent in the complicated group (*p* = 0.005). Of the 208 patients, 177 (85.1%) received conservative treatment, 7 (3.37%) had percutaneous abscess drainage, 8 (3.85%) underwent elective surgery, and 16 (7.69%) required emergency surgery. There were no differences in operative indications between complicated and uncomplicated diverticulitis.

The mean BMI of patients was determined to be 29.1 ± 5 kg/m^2^ (Table 2). The mean BMI of the complicated diverticulitis group (28.3 ± 5.05) was statistically significantly lower compared to the uncomplicated group (29.8 ± 4.88) (*p* = 0.037). Similarly, the VFA of the complicated group (179 ± 82.8 cm^2^) was found to be statistically significantly lower compared to the VFA of the uncomplicated group (203 ± 86.3 cm^2^) (*p* = 0.046). The mean SFA of the patients was 234 (± 96.4) cm^2^. The V/S ratio was calculated as 0.89 (± 0.44). There was no statistically significant difference between the complicated and uncomplicated groups in terms of SFA and V/S.

**Table 2:** Statistical Analysis of Body Mass Index, Visceral Fat Area, and Subcutaneous Fat Area of Complicated and Uncomplicated Groups Based on World Society of Emergency Surgery Classification

		All patients	Uncomplicated (WSES 0)	Complicated (WSES 1a-4)	p^a^
		*n* = 208	*n* = 111 (53.49%)	*n* = 97 (46.6%)	
BMI (kg/m^2^)					**0.037**
	Mean ± SD	29.1 ± 5	29.8 ± 4.88	28.3 ± 5.05	
	Median (min.–max.)	28.7 (16.6–47)	29.3 (21.6–45.9)	27.7 (16.6- 47)	
VFA (cm^2^)					**0.046**
	Mean ± SD	192 ± 85.3	203 ± 86.3	179 ± 82.8	
	Median (min.–max.)	189 (23.1–491)	199 (29.4–447)	180 (23.1–491)	
SFA (cm^2^)					0.264
	Mean ± SD	234 ± 96.4	242 ± 99.4	225 ± 92.6	
	Median (min.–max.)	219 (21.4–561)	230 (80.5–561)	209 (21.4–472)	
VFA/SFA					0.500
	Mean ± SD	0.89 ± 0.44	0.91 ± 0.45	0.87 ± 0.42	
	Median (min.–max.)	0.83 (0.15–2.86)	0.86 (0.15–2.86)	0.76 80.2–2.21)	

*Abbreviations: BMI *Body mass index, *VFA* Visceral fat area, *SFA *Subcutaneous fat area

^a^ Mann Whitney -U testi

*p<0.05, **p<0.01

When comparing patients undergoing conservative treatment with those undergoing interventional treatment, the BMI in the interventional treatment group (25.9 kg/m^2^) was found to be lower compared to the conservative treatment group (29 kg/m^2^) (*p* = 0.007) (Table 3). Similarly, a significant difference was found in terms of VFA between the interventional treatment group (156 cm^2^) and the conservative treatment group (193 cm^2^) (*p* = 0.025). There was no significant difference observed in terms of SFA and V/S ratio regarding treatment modalities.

**Table 3:** Comparison of conservative treatment and interventional treatment groups concerning body mass index (BMI), visceral fat area (VFA), subcutaneous fat area (SFA), and visceral-to-subcutaneous fat ratio (V/S)

		Conservative Treatment	Interventional treatment ***	p^a^
		*n* = 177 (85.1%)	*n* = 31 (14.9%)	
BMI (kg/m^2^)				0.007**
	Mean ± SD	29.4 ± 4.77	27.3 ± 6.01	
	Median (min.–max.)	29 (18.8–45.9)	25.9 (16.6–47)	
VFA (cm^2^)				0.025*
	Mean ± SD	196 ± 82.2	170 ± 100	
	Median (min.–max.)	193 (29.4–447)	156 (23.1–491)	
SFA (cm^2^)				0.419
	Mean ± SD	237 ± 96.4	215 ± 95.8	
	Median (min.–max.)	219 (76.7–561)	214 (21.4–449)	
VFA/SFA				0.659
	Mean ± SD	0.90 ± 0.44	0.85 ± 0.40	
	Median (min.–max.)	0.83 (0.15–2.86)	0.75 (0.21–2.13)	

***Interventional Treatment: Percutaneous drainage, Emergent and Elective Surgery

*Abbreviations: BMI-Body mass index, VFA- Visceral fat area, SFA-Subcutaneous fat area*

^a^ Mann Whitney -U test

*p < 0.05, **p < 0.01

The median BMI value (23.9) and VFA (142 cm^2^) of the 16 patients who underwent emergency surgery was 23.9, and it was were found to be statistically significantly lower compared to patients who did not undergo emergency surgery (29 kg/m^2^, 193 cm2, respectively) (*p* = 0.002, *p* = 0.034, respevtiveley) (Table 4). There was no significant difference between patients who underwent emergency surgery and those who did not in terms of SFA and V/S.

**Table 4:** Comparison of patients who underwent emergency surgery with other patients and Comparison of patients who underwent elective surgery with other patients concerning BMI, VFA, SFA, and V/S and

		Others	Emergency Surgery	p^a^	Others	Elective Surgery	p^a^
		*n* = 192 (92.3%)	*n* = 16 (7.7%)		*n* = 200 (96.16%)	*n* = 8 (3.84%)	
BMI (kg/m^2^)				0.002**			0.031*
	Mean ± SD	29.3 ± 4.85	26.4 ± 6.32		29.3 ± 4.97	25.1 ± 4.18	
	Median (min.–max.)	29 (16–45.9)	23.9 (22–47)		28.9 (23.1–491)	25.2 (16.6–30.2)	
VFA (cm^2^)				0.034*			0.043*
	Mean ± SD	195 ± 83.0	161 ± 107		194 ± 85.4	135 ± 63.9	
	Median (min.–max.)	193 (29.4–447)	142 (23.1–491)		191 (23.1–491)	112 (58.3–248)	
SFA (cm^2^)				0.240			0.288
	Mean ± SD	236 ± 95.0	205 ± 111		236 ± 97.2	191 ± 65.9	
	Median (min.–max.)	219 (76.7–561)	187 (21.4–449)		219 (21.4–561)	183 (78.5–268)	
VFA/SFA				0.926			0.404
	Mean ± SD	0.89 ± 0.44	0.87 ± 0.43		0.90 ± 0.44	0.76 ± 0.38	
	Median (min.–max.)	0.83 (0.15–2.86)	0.82 (0.21–2.139)		0.83 (0.15–2.86)	0.62 (0.39–1.38)	

*Abbreviations: BMI *Body mass index, *VFA* Visceral fat area, *SFA *Subcutaneous fat area

^a^ Mann Whityney -U test

*p < 0.05, **p < 0.01

The median BMI value (25.2 kg/m^2^) and and VFA (112 cm^2^)of the 8 patients who underwent elective surgery were significantly lower compared to who did not undergo elective surgery (28.9 kg/m^2^, 191 cm^2^, respectively) (*p* = 0.031, *p* = 0.043, respectively) (Table 4). There was no significant difference between patients who underwent elective surgery and those who did not in terms of SFA and V/S.

A more comprehensive analysis was also conducted where the complicated group (radiological and/or clinical) are comprised of those categorized as WSES Stage 1 A, 1B, 2 A, 2B, 3, and 4 at the time of admission, as well as cases presenting with obstruction or fistula, abscess formation during follow-up, emergency or elective surgeries, percutaneous abscess drainage, and postoperative complications (*n* = 94) (Table 5). Patients who did not have any of these complications were classified into the non-complicated group (*n* = 114). The median BMI value of patients in the complicated group (27.3 kg/m^2^) was significantly lower than the non-complicated group (29.5 kg/m^2^) (*p* = 0.006). Similarly, there was a significant difference in VFA examination between the two groups (179 cm^2^, 201 cm^2^ respectively, *p* = 0.033) There was no significant difference in SFA and V/S between the two groups.

**Table 5:** Comparison of patients exhibiting radiological and/or clinical complications at presentation, and those who developed complications during follow-up, with Non-complicated patients regarding BMI, VFA, SFA, and V/S

		Non-Complicated***	Complicated(Radiological and/or Clinical)****	p^a^
		*n* = 114 (54.8%)	*n* = 94 (45.2%)	
BMI (kg/m^2^)				0.006**
	Mean ± SD	29.9 ± 4.85	28.1 ± 5.02	
	Median (min.–max.)	29.5 (21.5–45.9)	27.3 (16.6–47)	
VFA (cm^2^)				0.033*
	Mean ± SD	203 ± 85.2	178 ± 83.8	
	Median (min.–max.)	201 (29.4–447)	179 (23.1–491)	
SFA (cm^2^)				0.212
	Mean ± SD	243 ± 98.0	223 ± 93.9	
	Median (min.–max.)	220 (80.5–561)	212 (21.4–468)	
VFA/SFA				0.401
	Mean ± SD	0.92 ± 0.45	0.86 ± 0.42	
	Median (min.–max.)	0.87 (0.15–2.86)	0.76 (0.21–2.219	

****Complicated(Radiological and/or Clinical)**** Patients categorized as WSES Stage 1 A, 1B, 2 A, 2B, 3, and 4 at the time of admission, as well as cases presenting with obstruction or fistula, abscess formation during follow-up, emergency or elective surgeries, percutaneous abscess drainage, and postoperative complications

*** Non-Complicated: Patients with no radiological or clinical complications

*Abbreviations: BMI* Body mass index, *VFA* Visceral fat area, *SFA *Subcutaneous fat area

^a^ Mann Whityney -U test

*p < 0.05, **p < 0.01

Univariate Cox proportional hazards regression analysis identified several clinical, laboratory, radiologic and demographic factors significantly associated with the risk of complicated diverticulitis, including VFA (HR: 1.005, *p* = 0.0009), SFA (HR: 0.998, *p* = 0.0061), V/S ratio (HR: 3.40, *p* = 0.0002), CRP (HR: 1.021, *p* < 0.0001), WBC count, neutrophil metrics, procalcitonin, bilirubin levels, male gender, age ≥ 60, multiple episodes of diverticulitis, and sigmoid colon localization (Table 6).

**Table 6:** Univariate and Multivariate Analysis of Predictors of Uncomplicated versus Complicated Diverticulitis

	Uncomplicated Diverticulitis	Complicated Diverticulitis	Univariate			Multivariate		
	(WSES 0)	(WSES 1a-4)	HR*	95% CI*	P Value	HR*	95% CI*	*P* Value
	*n* = *111 (53.49%)*	*n* = *97 (46.6%)*						
Age ≥ 60	49 (44.14%)	43 (44.32%)	13.299	1.0251–1.7252	**0.0315**	1.15	0.98–1.35	0.0917
Male Gender	53 (47.74%)	46 (47.44%)	12.735	1.0677–1.5188	**0.0076**	1.3	1.06–1.6	**0.0129**
Multiple Episodes	30 (27.02%)	38 (39.17%)	16.361	1.248–2.1443	**0.0004**	1.42	1.07–1.88	**0.0145**
Diverticulitis Localization (Sigmoid Colon)	74 (66.66%)	80 (82.47%)	0.8043	0.673–0.9609	**0.0142**	0.84	0.71–0.99	**0.0451**
C-Reactive Protein (mg/dl) > 8.9	31 (27.92%)	47 (48.45%)	1.021	1.0143–1.028	**0**	1.014	1.004–1.0023	**0.0089**
Procalcitonin (ng/ml) > 0.28	41 (45.55%)	60 (69.76%)	10.219	1.0018–1.0423	**0.0324**	1.0029	0.9997–1.0062	0.0847
White Blood Cell (WBC) Count (× 10^3^/µL) > 12.5	41 (36.93%)	48 (49.48%)	10.001	1–1.0002	**0.0023**	-	-	> 0.05
Neutrophil Percentage (%) > 76.5	41 (36.93%)	58 (59.79%)	10.234	1.0144–1.0325	**0**	-	-	> 0.05
Neutrophil Count (× 10^3^/µL) > 10.3	32 (28.82%)	40 (41.23%)	10.001	1–1.0001	**0.0032**	-	-	> 0.05
Total Bilirubin (mg/dl) > 0.91	47 (42.34%)	52 (53.60%)	16.253	1.3758–1.9191	**0**	1.24	1.01–1.53	**0.0382**
Direkt Bilirubin (mg/dl) > 0.28	46 (41.44%)	57 (58.76%)	21.688	1.4163–3.3203	**0.0003**	-	-	> 0.05
Visceral Fat Area (VFA)(cm^2^) < 170	60 (56.07%)	45 (46.87%)	10.054	1.0022–1.0086	**0.0009**	-	-	> 0.05
Subcutaneous Fat Area (SFA) (cm^2^) < 230	58 (54.20%)	41 (42.70%)	0.9987	0.9971–0.9998	**0.0061**	-	-	> 0.05
VFA/SFA Ratio (V/S) < 0.68	70 (65.42%)	59 (61.45%)	33.994	1.7648–6.5534	**0.0002**	2.64	1.44–4.84	**0.0021**
Body Mass Index (BMI) (kg/cm^2^) < 28.4	65 (59.63%)	43 (45.74%)	10.142	0.9942–1.0346	0.2097	-	-	-

*HR* Hazard Ratio. *CI* (Confidence Interval)

In the multivariable Cox regression model, independent predictors of complicated diverticulitis included:

V/S Ratio (HR: 2.64, 95% CI: 1.44–4.84, *p* = 0.0021), CRP (HR: 1.014, 95% CI: 1.004–1.023, *p* = 0.0089), Total Bilirubin (HR: 1.24, 95% CI: 1.01–1.53, *p* = 0.0382), Multiple Episodes (HR: 1.42, 95% CI: 1.07–1.88, *p* = 0.0145), Male Gender (HR: 1.30, 95% CI: 1.06–1.60, *p* = 0.0129) and Sigmoid Colon Localization (HR: 0.84, 95% CI: 0.71–0.99, *p* = 0.0451) (Table 6). VFA lost significance in multivariate analysis (*p* = 0.0847), likely due to collinearity with the V/S ratio, while variables such as age and procalcitonin also became insignificant. These results indicate that fat distribution, inflammatory markers, number of episodes, and demographic factors are critical independent risk factors for complicated diverticulitis.

## Discussion

In our study, we explored the relationship between visceral adiposity, and complications of acute diverticulitis. Contrary to the findings of several studies, the mean VFA of the 97 patients with complicated diverticulitis was significantly lower than 111 patients of the uncomplicated group. This was further supported by the finding that the interventional treatment group exhibited notably lower VFA and BMI compared to the conservative treatment group. Patients undergoing elective or emergency surgery also showed significantly lower VFA than those who did not have surgery. When analyzing all radiologically and/or clinically complicated patients against the remaining patients, the complicated group had significantly lower VFA and BMI. However, no significant differences were observed in SFA or the V/S ratio. These results suggest that lower visceral adiposity measurements may be linked to an increased occurrence of complications in diverticular disease, diverging from some of the current literature.

Jeong et al. conducted a retrospective analysis of 140 patients and found significantly higher VFA in those with complicated diverticulitis compared to those without, although no significant difference in BMI was observed [19]. Docimo’s retrospective analysis of 32 elective versus 34 emergency surgery patients for diverticulitis revealed significantly higher VFA and V/S ratios in the emergency group, with no notable BMI differences [22]. Another analysis of 352 patients found no significant differences in VFA, SFA, total fat area, or V/S ratios between complicated and uncomplicated cases. Additionally, emergency surgery likelihood increased in patients with a V/S ratio less than 0.4 [27].

In our study, the average BMI of patients with complicated diverticulitis was significantly lower than that of those with uncomplicated cases. This trend extended to patients requiring radiological or surgical interventions, who also exhibited lower BMIs compared to those treated conservatively. A prospective study of 628 patients with diverticular disease revealed that overweight and obese individuals had higher hospitalization risks compared to those with normal weight [18]. Obese women were twice as likely to develop perforation or abscess, while women with low BMI also faced increased risks. Strate et al. found a significant link between high BMI and the risk of diverticulitis and bleeding [16]. A meta-analysis of six studies found that a 5-unit increase in BMI raises the relative risk of diverticulitis by 31% and complications by 20%, with non-linear risks observed even in individuals with low BMI [29]. While many studies suggest a linear relationship between increased BMI and diverticulitis, others, including ours, indicate otherwise. Lee et al. found that the diverticulitis group had a significantly lower BMI than both the diverticulosis and control group [26]. Additionally, Sagiroglu’s multicenter study revealed that patients with recurrent diverticulitis had lower BMIs compared to those with a single episode, with no surgeries necessary among obese or morbidly obese individuals [28].

In our study lower V/S ratio was the strongest independent risk factor for developing complicated diverticulitis according to multivariate Cox regression analysis. Furthermore, male gender, multiple number of episodes, sigmoid colon localization, CRP, Total Bilirubin were also found to be independent predictors. Diverticulitis localized to the sigmoid colon was associated with a lower risk of complications, suggesting that disease in this location may follow a more indolent course compared to other segments of the colon. In the multivariate analysis VFA and SFA did not demonstrate efficacy in differentiating complicated disease. The non-significance of VFA and SFA reflects the complex interplay of interrelated predictors and the statistical necessity for each variable to demonstrate independent, non-redundant predictive value within a multivariate framework. Lower VFA showed a statistical association with complicated disease in unadjusted comparisons but did not function as an independent risk factor in multivariable analysis. These findings highlight the complex, potentially non-linear relationship between adipose tissue and disease severity. They emphasize the need for further research into fat quality, inflammatory signaling, and the host immune response in the pathogenesis of complicated diverticulitis.

Inconsistent literature on the relationship between diverticulitis, its complications, visceral adiposity, and BMI may be explained by the"obesity paradox,"identified in 2008. This paradox suggests that increased BMI post-surgery may not affect mortality and morbidity negatively, possibly offering a protective effect. Studies indicate that lower BMI groups may actually have higher risks [30–32]. Notably, a study of 101,078 patients showed significantly lower mortality among overweight and obese individuals following emergency surgery compared to those with normal BMI, reinforcing the protective role of obesity [33]. Furthermore, adipose tissue is believed to modulate IL-6 and TNF receptors, exhibiting anti-inflammatory properties, while a meta-analysis of 55,391 patients in China found high BMI beneficial for colorectal cancer outcomes [34]. The majority of research examining the link between obesity and diverticular disease has been retrospective and has exclusively utilized BMI as a measure of obesity. In studies that incorporate VFA, the small sample size and lack of multivariate analyses may hinder the ability to draw conclusive insights.

The secondary endpoints of our study include the identification of inflammatory markers, disease localization, demographic variables and clinical progression. According to the WSES classification, the complicated diverticulitis group exhibited significantly elevated levels of CRP, WBC, neutrophil count, neutrophil percentage, procalcitonin, and direct bilirubin in comparison to the non-complicated group. Additional research corroborates that parameters such as procalcitonin and CRP serve as crucial markers for differentiating between complicated and uncomplicated diverticulitis. A particular study indicated that isolated hyperbilirubinemia may occur in diverticular perforation cases without a concurrent increase in liver enzymes [11, 35–40]. Furthermore, the length of hospitalization was notably longer in the complicated group. Interventional treatments, including percutaneous drainage, as well as both emergency and elective surgeries, were significantly more prevalent in the complicated cohort. Recent prospective observational research has revealed statistically significant differences among WSES stages concerning clinical and analytical data, treatment decisions, and outcomes, thus affirming the substantial validity of the WSES classification in disease assessment[41].

Our study has several limitations. While the number of patients is sufficient for a single center, there are studies in the literature conducted with larger cohorts. However, compared to prospective studies, we can say that our study provides a comprehensive analysis in terms of both the number of patients and the parameters examined. However, while there is enough data to compare the complicated and uncomplicated diverticulitis groups, the limited number of subgroups within the complicated category might have hindered accurate analysis of these subgroups. Additionally, due to only a small portion of patients with diverticular disease experiencing diverticulitis and those with diverticulitis requiring surgery at relatively low rates, the number of patients undergoing surgery is also low. Another limitation is that CT-based measurements are influenced by slice selection and patient positioning, impacting their interpretation. Variability in anatomical slices can lead to discrepancies, as thicker slices may include subcutaneous fat, while thinner slices may miss visceral fat. The positioning of the patient influences fat distribution, and movements during the scanning process may introduce artifacts that further hinder the clarity of the data. These factors necessitate caution in interpreting VFA data and highlight the need for standardized imaging protocols to enhance measurement reliability and clinical relevance. Furthermore, while comparing the relationship between visceral adiposity and BMI with diverticulitis complications using the WSES classification is a strong aspect of our study, the factors causing complications of diverticular disease and its pathogenesis have not been clearly identified in the literature, indicating a need for more comprehensive studies.

## Conclusion

In conclusion, our study confirms that complicated diverticulitis cases have lower BMI and VFA, and V/S < 0.68 is highly predictive in these patients. While there is a correlation between increased BMI or obesity and the incidence of colonic diverticular disease, this correlation appears to be inversely related to complicated diverticular disease. While diverticula are often seen more frequently in obese individuals, we propose that an increase in visceral adipose tissue might actually provide a protective effect by restricting the development of these diverticula into more complicated forms. However, larger patient cohorts and multicenter prospective studies are needed to investigate the factors affecting diverticular disease complications, along with multivariate analysis of various biomarkers in different stages of complicated diverticulitis.

## Data Availability

Data is provided within the manuscript or supplementary information files.
